# Paracetamol (acetaminophen) rescues cognitive decline, neuroinflammation and cytoskeletal alterations in a model of post-operative cognitive decline (POCD) in middle-aged rats

**DOI:** 10.1038/s41598-021-89629-y

**Published:** 2021-05-12

**Authors:** B. Garrone, L. Durando, J. Prenderville, E. Sokolowska, C. Milanese, F. P. Di Giorgio, C. Callaghan, M. Bianchi

**Affiliations:** 1 Angelini Pharma S.p.A., Viale Amelia, 70, 00181 Rome, Italy; 2 Transpharmation Ireland Ltd., Trinity College Dublin-Institute of Neuroscience (TCIN), Lloyd Institute, Trinity College, Dublin 2, Ireland; 3 Ulysses Neuroscience Ltd, Room 3.57B, Trinity College Dublin-Institute of Neuroscience (TCIN), Lloyd Institute, Trinity College, Dublin 2, Ireland

**Keywords:** Neurology, Neuroscience, Learning and memory, Molecular neuroscience, Neural ageing, Neuroimmunology, Neurotrophic factors, Regeneration and repair in the nervous system, Synaptic plasticity

## Abstract

Post-operative cognitive dysfunction (POCD) is a debilitating clinical phenomenon in elderly patients. Management of pain in elderly is complicated because analgesic opiates elicit major side effects. In contrast, paracetamol (acetaminophen) has shown analgesic efficacy, no impact on cognition, and its side effects are well tolerated. We investigated the efficacy of paracetamol, compared to the opioid analgesic buprenorphine, in a model of POCD by investigating cognitive decline, allodynia, peripheral and hippocampal cytokines levels, and hippocampal microtubule dynamics as a key modulator of synaptic plasticity. A POCD model was developed in middle-aged (MA) rats by inducing a tibia fracture via orthopaedic surgery. Control MA rats did not undergo any surgery and only received isoflurane anaesthesia. We demonstrated that cognitive decline and increased allodynia following surgery was prevented in paracetamol-treated animals, but not in animals which were exposed to anesthesia alone or underwent the surgery and received buprenorphine. Behavioral alterations were associated with different peripheral cytokine changes between buprenorphine and paracetamol treated animals. Buprenorphine showed no central effects, while paracetamol showed modulatory effects on hippocampal cytokines and markers of microtubule dynamics which were suggestive of neuroprotection. Our data provide the first experimental evidence corroborating the use of paracetamol as first-choice analgesic in POCD.

## Introduction

Post-operative cognitive dysfunction (POCD) is a recognized clinical phenomena defined as a new cognitive impairment arising after anaesthesia and surgery with a higher prevalence in elderly patients^1^. The available data from animal research suggest that POCD has a multifactorial pathogenesis resulting from a combination of anaesthesia and surgery effects on the systemic immune system^2^, neuroinflammation^3,4^, and synaptic plasticity^5,6^. Currently, no treatment is available to either prevent or rapidly treat POCD symptomatology, despite global population aging and extensive new developments in health care which both imply increasing incidences of surgery in older patients. The most common POCD symptomatology is memory impairment, with patients showing impaired performance on cognitive tasks^7^. POCD has been documented in 41.4% of elderly patients (aged ≥ 60) who had undergone any major surgery^8^. It is also shown that at 3 months after surgery, POCD can still be present in 12.7% of elderly patients^8^.

Orthopaedic surgery is highly associated with POCD^8^ and this category of surgery is well known to be accompanied by significant acute post-operative pain^9^. Management of pain in aged patients with cognitive decline has to be approached with caution since the use of analgesic opiates, for example, elicits side effects on cognition and bowel function and may precipitate delirium^10^. In contrast, paracetamol (acetaminophen; Par) has shown analgesic efficacy, no impact on cognition and well tolerated side effects^10^. Par mechanism of action is still not fully understood, the drug lacks peripheral anti-inflammatory properties and it passes the blood–brain barrier to be homogenously distributed throughout the CNS even at low doses^11–13^. Experimental evidence suggest that the analgesic effects of Par might be due inhibition of Cyclooxygenase (COX) in the brain^14^; and indirect activation of cannabinoid CB1 receptor^15,16^. Furthermore, growing evidence shows that Par have neuroprotective effects both in vitro^17^ and in vivo^18^.

The current study examined the efficacy of Par (75 mg/kg and 150 mg/kg; i.p.) in rescuing behavioural and molecular alterations induced by a model of orthopaedic surgery in middle-aged (MA) rats compared to isoflurane anaesthesia and the opioid analgesic buprenorphine (Bup; 0.05 mg/kg and 0.1 mg/kg; s.c.). The delayed non-match-to-sample (DNMTS) operant task was used to measure short-term working memory and flexibly modulating behaviour in rats through time (i.e. pre- and post-surgery) following pharmacological treatments. Pain was measured using the cold plate test on day 1–5 post surgery in order to evaluate thermal allodynia. Additionally, a panel of cytokines was analysed in plasma and hippocampus in line with the neuroinflammatory hypothesis of POCD^3^. Further analysis was conducted to explore neuroprotective effects of paracetamol on microtubule dynamics, a key modulator of synaptic plasticity involved in learning and memory^19^. Thus, α-tubulin PTMs related to the C-terminal detyrosination/tyrosination cycle, such as tyrosination (Tyr-Tub: associated with dynamic microtubules), detyrosination (Glu-Tub: associated with stable microtubules), deglutamylation (Δ2-Tub: neuronal specific) were analysed in the hippocampus.

## Results

### Validation and identification of age-related memory impairments in the DNMTS assay

**Figure 1:**
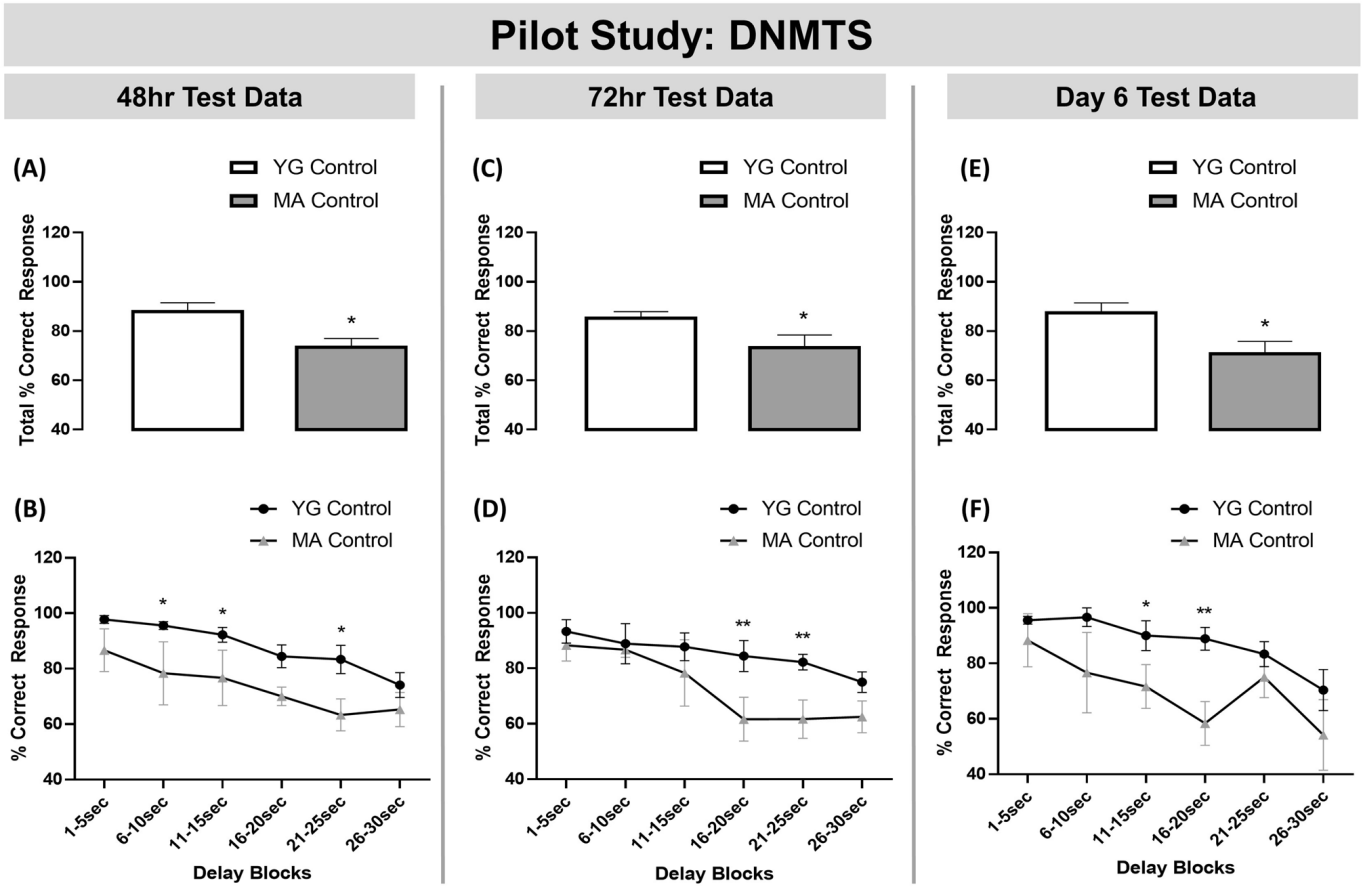
MA rats were impaired in the DNMTS task compared to YG controls. MA animals were impaired in the DNMTS task compared to YG controls at: (**A**) 48 h test-point overall performance; (**B**) 48 h test-point at the 6–10 s, 11–15 s to 21–25 s delay time bins; (**C**) 72 h test-point overall performance; (**D**) 72 h test-point at the 16–20 s and 21–25 s delay time bins; (**E**) Day 6 test-point overall performance; and (**F**) Day 6 test-point at the 11–15 s and 16–20 s delay time bins. Histograms represent Total % Correct response (the mean group performance across all delay lengths in the DNMTS task ± SEM), n = 4–6 per group. DNMTS trials were sorted by performance according to length of delay on individual trials and were grouped according to 5-s intervals (1–5, 6–10, 11–15, 16–20, 21–25, and 26–30) represented by line graphs (mean ± SEM). Two-way ANOVA followed by Fishers LSD analysis **p < 0.01, *p < 0.05 *vs.* YG controls.

To corroborate previous findings, we first conducted a pilot study to demonstrate the natural delay-induced cognitive impairment in MA rats compared to young (YG) controls. MA rats were impaired in the DNMTS paradigm, compared to YG controls at each test point; 48 h, 72 h and Day 6 of testing (Fig. 1A,C,E respectively; p < 0.05 for each). The delayed-induced group differences in the DNMTS task were analysed by two-way ANOVA for repeated measures. ANOVA analysis yielded a significant main effect of age [F_(1, 8)_ = 7.6; p = 0.02] and delay [F_(5, 40)_ = 8.2, p < 0.0001] and no significant interaction at 48 h test point. Fisher's LSD analysis revealed that the MA group was significantly impaired at the following delay time bins; 6–10 s (p < 0.05), 11–15 s (p < 0.05) and 21–25 s (P < 0.05) (Fig. 1B). At 72 h test point, the ANOVA analysis yielded a significant main effect of age [F_(1, 8)_ = 4.6; p = 0.05] and delay [F_(5, 40)_ = 6.8, p < 0.0001]. Fisher's LSD analysis revealed the MA group were significantly impaired at the longer delay time bins at 16–20 s and 21–25 s (Fig. 1D: p < 0.01 and p < 0.01, respectively). ANOVA analysis yielded a significant main effect of age [F_(1, 8)_ = 5.1; p = 0.05] and delay [F_(5, 40)_ = 7.5, p < 0.0001] at 6 days test point. Fisher's LSD analysis revealed the MA group were significantly impaired at the 11–15 s time bin and the 16–20 s time bin (Fig. 1F: p < 0.05 and p < 0.01, respectively).

### Paracetamol has a significant impact on the extent of POCD

**Figure 2:**
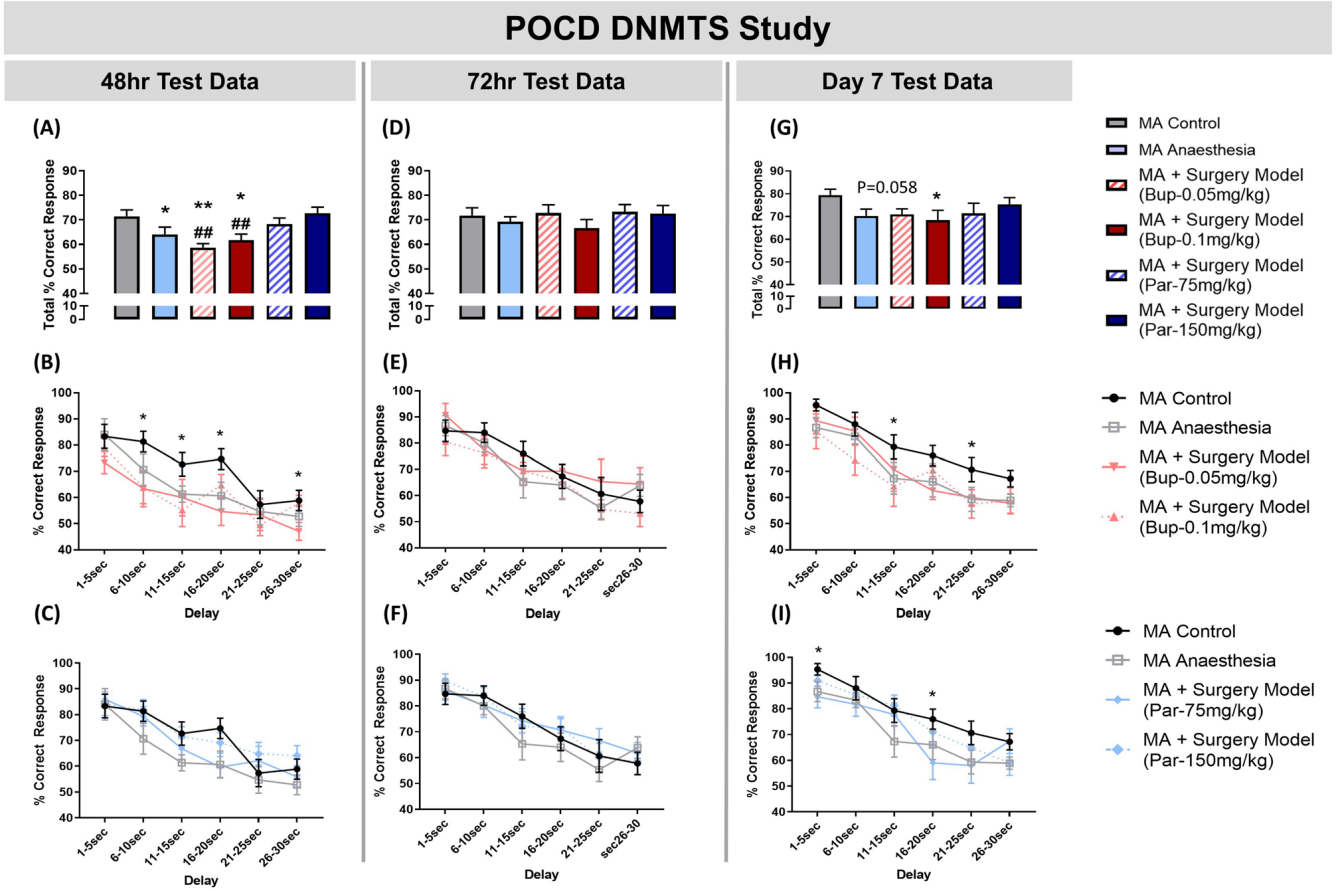
Paracetamol (Par) treatment appears to recover POCD in the DNMTS. Results at 48 h test-point showed: (**A**) MA rats exposed to anaesthetic only or which underwent surgery (MA + Surgery Model) treated with Buprenorphine at 0.05 mg/kg, i.p. or 0.1 mg/kg, i.p. were impaired in the DNMTS task compared to MA controls. In contrast, MA animals treated with Par-75 mg/kg, s.c. or Par-150 mg/kg, s.c. were not impaired in the task compared to MA controls; (**B**) Examination of the Bup groups compared to control groups across delay length showed that surgery model groups treated with Bup-0.05 mg/kg or Bup-0.1 mg/kg were impaired at 6–10 s, 11–15 s, 16–20 s and 26–30 s time bins; (**C**) Examination of the Par groups compared to control groups across delay length showed that surgery model groups treated with Par-75 mg/kg or Par-150 mg/kg did not have any delay-induced impairment. Results at 72 h test-point showed: (**D**) No differences were observed between groups; (**E**) and (**F**) No group differences were observed following examination of performance across different delay lengths. Results at Day 7 test-point showed: (**G**) MA animals which underwent the surgery model treated with Bup-0.1 mg/kg were significantly impaired compared to MA Controls. Par treatment (75 mg/kg or 150 mg/kg) to surgery model animals prevented long lasting cognitive impairments; (**H**) MA + Surgery Model groups treated with Bup (0.05 mg/kg or 0.1 mg/kg) were impaired at delay time lengths 11–15 s to 21–25 s compared to MA control; (**I**) MA + Surgery Model group treated with Par-75 mg/kg were impaired at delay lengths 1–5 s and 16–20 s compared to MA control. Histograms represent Total % Correct response (the mean group performance across all delay lengths in the DNMTS task ± SEM), n = 5–11 per group. DNMTS trials were sorted by performance according to length of delay on individual trials and were grouped according to 5-s intervals (1–5, 6–10, 11–15, 16–20, 21–25, and 26–30) represented by line graphs (mean ± SEM). Two-way ANOVA followed by Fisher’s LSD analysis **p < 0.01, *p < 0.05 *vs.* MA control; ^##^p < 0.01 vs. MA + Surgery Model (Par-150 mg/kg) group.

Following validation of in the DNMTS task in MA rats, we compared the effects of the post-operative analgesics treatment with Par or Bup on performance in the DNMTS task. Par was used at two doses (75 mg/kg; i.p. and 150 mg/kg; i.p.) and compared to Bup at two doses (0.05 mg/kg; s.c. and 0.1 mg/kg; s.c.). First testing in the DNMTS task resumed 48 h following surgery/control procedures. All drugs were administered daily at 23 h before test. Animals exposed to anaesthetic alone or animals which underwent surgery and received Bup at either dose were impaired in total % correct response in the DNMTS task (Fig. 2A, p < 0.05, p < 0.01, p < 0.05, respectively). Further, surgery model groups treated with Bup-0.05 mg/kg or Bup-0.1 mg/kg were significantly impaired in the DNMTS compared to the surgery group treated with the Par doses (Fig. 2A, p < 0.01 for both). Analysis by two-way ANOVA for repeated measures revealed a significant effect of analgesia treatment [F_(5, 47)_ = 3.71; p = 0.006] and delay [F_(5, 235)_ = 31.3; p < 0.0001] with no significant interaction. Fisher's LSD analysis further showed that the surgical group treated with Bup at either dose were significantly impaired at both long and short time bins compared to MA controls (Fig. 2B). In contrast, the Par-treated surgery groups were not impaired, compared to the MA Controls, at any delay point (Fig. 2C). No group differences were observed at the 72 h test point (Fig. 2D–F). When animals were tested at 7 days post-surgery/control procedures, only the surgery groups treated with Bup were still impaired in overall total performance in the DNMTS task compared to MA Controls (Fig. 2G, p < 0.05 for Bup-150 mg/kg, p = 0.058 for Bup-75 mg/kg). The two-way ANOVA for repeated measures did not reveal a significant overall effect of analgesia treatments [F_(5, 47)_ = 1.4; p = 0.2] but there was a significant effect of delay [F_(5, 235)_ = 53.3; p < 0.0001] with no significant interaction. However, Fisher’s LSD pairwise comparisons revealed the surgical group treated with Bup at either dose were significantly impaired compared to MA controls (Fig. 2H) at 11–15 s delays (p < 0.05 for both) and at 21–25 s delays (p < 0.05, for both). The surgical group treated with Par-75 mg/kg were impaired at earlier time bins such as at 1–5 s delays (p < 0.05) and 11–16 s delays (p < 0.05) compared to MA Controls (Fig. 2I).

### Buprenorphine treatment precipitates negative effects on cognition following surgery.

**Figure 3:**
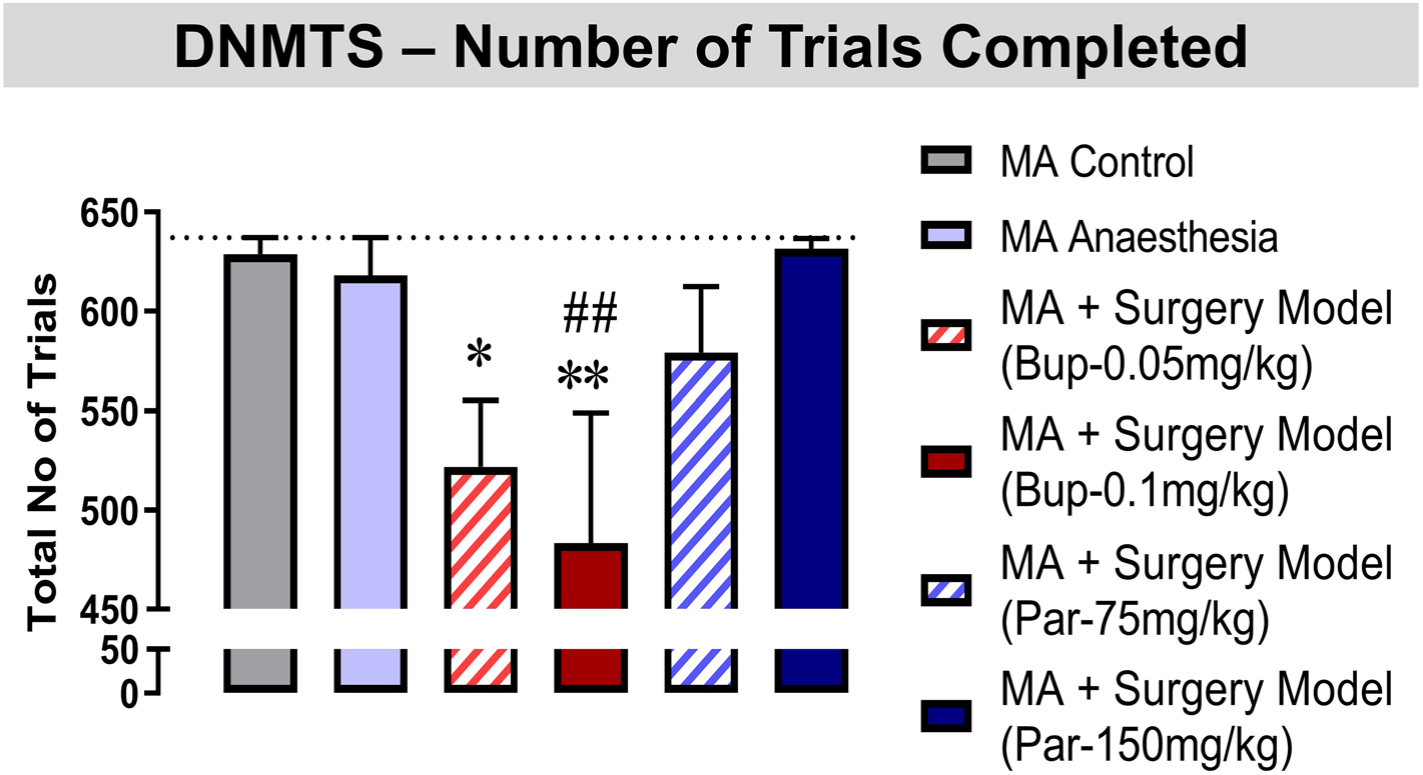
Buprenorphine (Bup) treatment precipitates negative effects on cognition following surgery. Animals which underwent the surgery model procedure and received buprenorphine (Bup, s.c.) treatment were impaired in their ability to complete DNMTS trials over the week of test days compared to animals treated with Paracetamol (Par, i.p.). Animals which did not complete the necessary 90 trials per test session where not included in the data analysis. Histograms represent the number of trials completed over all test sessions. One-way ANOVA followed by Fisher’s LSD analysis **p < 0.01, *p < 0.05 *vs.* MA control; ^##^p < 0.01 *vs.* MA anaesthesia group.

All groups showed a small amount of attrition across the DNMTS task, with 1–3 rats from the MA Control, MA + Anaesthesia, and the two Par-treated MA + Surgery groups being excluded from the final analysis as they failed to complete the required number of trials in the task. However, Bup saw much greater levels of attrition with 7 and 6 rats being excluded from the MA + Surgery (Bup-0.05 mg/kg) and MA + Surgery (Bup-0.1 mg/kg) groups respectively. One-way ANOVA analysis of total number of trials completed yielded significant group differences [F_(5, 64)_ = 3.182, p = 0.0126]. Fishers LSD revealed that MA animals which underwent surgery and were treated with Bup-0.05 mg/kg or Bup-0.1 mg/kg completed significantly less trials than the MA control group (Fig. 3: p < 0.05 and p < 0.01 respectively). Furthermore, animals which received Bup-0.1 mg/kg completed significantly less trials than the MA anaesthesia alone group (p < 0.01), suggesting Bup-0.1 mg/kg further impaired cognitive abilities. Animals receiving Par-75 mg/kg or Par-150 mg/kg were not impaired in trials completed compared to the MA control or MA Anaesthesia group.

### Paracetamol has long-lasting analgesic effect in the cold plate test following surgery

**Figure 4:**
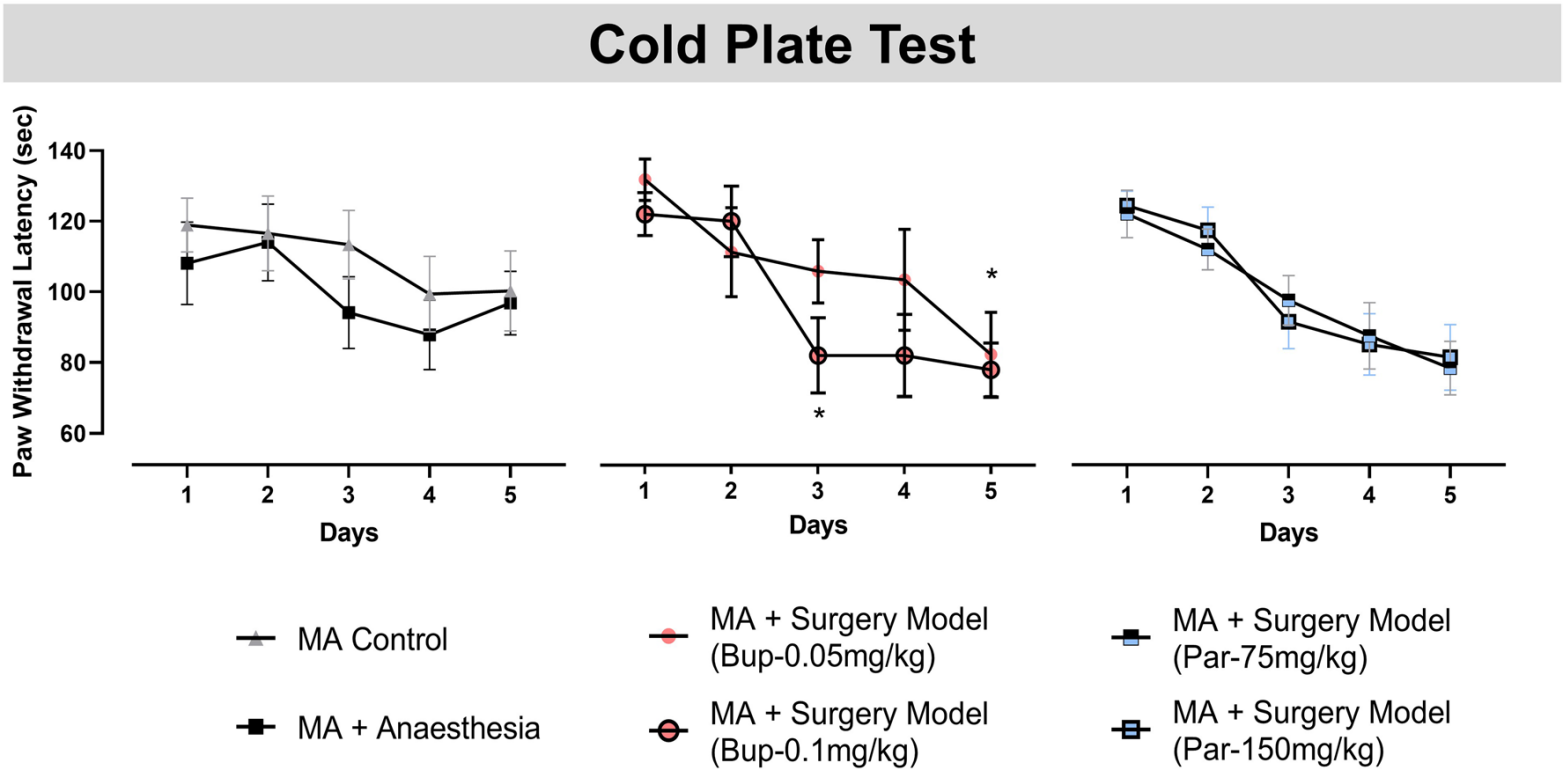
Paracetamol (Par) has long-lasting analgesic efficacy in the cold plate allodynia test. Performance in the cold plate test was assessed on days 1 to 5 post-surgery/control procedure. MA surgery model groups treated with Bup-0.05 mg/kg or Bup-0.1 mg/kg (s.c.) displayed increased sensitivity compared to MA Controls on test days 3 and 5. Line graph represent mean performance over 5 test days (mean ± SEM), n = 11–12. Two-way ANOVA followed by Fisher’s LSD analysis **p < 0.01, *p < 0.05 vs. MA control.

In addition to examination of cognition we also looked at the long-lasting effects of analgesia treatments in the cold plate test on days 1 to 5 post-surgery/control procedure. All drugs were administered daily 24 h before test in the cold plate assay. Interestingly, Par-75 mg/kg and Par-150 mg/kg was more effective at alleviating sensitivity in the cold plate test than Bup. Analysis by two-way ANOVA for repeated measures yielded significant main effect of test days [F_(4, 188)_ = 18.17; p < 0.0001]. Fisher’s LSD revealed that the surgery group treated with the Bup-0.5 mg/kg were significantly more sensitive on day 5 compared to the MA control group. In addition, Fisher’s LSD pairwise comparisons revealed that the surgery group treated with Bup-0.1 mg/kg dose were significantly more sensitive on day 3 and day 5 compared to the MA control group (Fig. 4). Importantly, animals treated with Par-75 mg/kg or Par-150 mg/kg, were not more sensitive in the cold plate test compared to MA controls.

### Par and Bup have differential effects on peripheral cytokine levels following surgery.

Plasma cytokine levels were measured following sample collection at the end of the behavioural experiments, i.e. 8 days after exposure to isoflurane anaesthesia or undergoing surgery procedure with analgesia treatments. Complete results are presented in Table 1. Briefly, Fisher’s LSD test revealed that MA animals which underwent surgery and were treated with Par-150 mg/kg or Par-75 mg/kg had significantly higher levels of TNF-α compared to MA control (Table 1: p < 0.05 for both). IL-10 levels were significantly increased in MA animals which underwent surgery and were treated with Bup-0.1 mg/kg compared to MA animals exposed to anaesthesia only (Table 1: p < 0.05). Moreover, IL-13 levels were significantly increased in MA animals which underwent surgery and were treated with Bup-0.1 mg/kg compared to MA animals exposed to anaesthesia only (Table 1: p < 0.05) and MA control animals (Table 1: p < 0.01). IL-6 levels were significantly increased in MA animals which underwent surgery and were treated with Bup-0.1 mg/kg compared to MA controls (Table 1: p < 0.05). Interestingly, MA animals which underwent the surgery model and were treated with Bup-0.05 mg/kg or Bup-0.1 mg/kg had significantly higher plasma IL-5 levels compared to MA controls (p < 0.05 and p < 0.01, respectively).

**Table 1:** Analysis of circulating cytokine levels in plasma.

Plasma cytokines	MA control	MA + anaesthesia	MA + surgery model (Bup-0.05 mg/kg)	MA + surgery model (Bup-0.1 mg/kg)	MA + surgery model (Par-75 mg/kg)	MA + surgery model (Par-150 mg/kg)	F value
**Pro-inflammatory**							
TNF-α	100 ± 4.9 n = 9	112 ± 9 n = 10	135 ± 17 n = 5	117 ± 10 n = 7	227 ± 65* n = 8	241 ± 63*^,#^ n = 11	F_(5, 44)_ = 2.258 p = 0.0651
IFN-γ	100 ± 46 n = 10	55 ± 13 n = 10	35 ± 20 n = 5	64 ± 14 n = 7	50 ± 13 n = 10	67 ± 14 n = 11	F_(5, 47)_ = 0.6873 p = 0.6354
IL-1β	100 ± 12 n = 4	150 ± 45 n = 5	165 ± 67 n = 5	225 ± 114 n = 4	134 ± 35 n = 7	195 ± 35 n = 8	F_(5, 27)_ = 0.6073 p = 0.6949
IL-6	100 ± 11 n = 9	230 ± 81 n = 11	157 ± 43 n = 4	330 ± 116* n = 7	150 ± 34 n = 9	166 ± 29 n = 11	F_(5, 45)_ = 1.517 p = 0.2036
IL-5	100 ± 16 n = 6	136 ± 60 n = 7	498 ± 99* n = 4	638 ± 276** ^##^ n = 5	318 ± 63 n = 11	359 ± 97 n = 10	F_(5, 37)_ = 2.810 p = 0.0300
KC/GRO (CXCL1)	100 ± 8 n = 9	102 ± 9 n = 10	81 ± 6 n = 5	85 ± 6 n = 7	114 ± 24 n = 9	129 ± 44 n = 11	F_(5, 45)_ = 0.4344 p = 0.8222
**Anti-inflammatory**							
IL-10	100 ± 6 n = 9	120 ± 13 n = 10	99 ± 6 n = 4	153 ± 29* n = 7	102 ± 12 n = 9	131 ± 16 n = 11	F_(5, 44)_ = 1.567 p = 0.1892
IL-13	100 ± 6 n = 9	137 ± 39 n = 10	220 ± 102 n = 4	278 ± 69**^,#^ n = 7	119 ± 24 n = 9	125 ± 20 n = 11	F_(5, 44)_ = 2.752 p = 0.0301
IL-4	100 ± 9 n = 9	116 ± 14 n = 10	92 ± 12 n = 5	151 ± 37 n = 7	95 ± 10 n = 9	135 ± 17 n = 11	F_(5, 45)_ = 1.535 p = 0.1982

Data are expressed as a percentage of the MA Control group (mean ± SEM), n = 5–11 per group. One-way ANOVA followed by Fisher’s LSD analysis **p < 0.01, *p < 0.05 *vs.* MA control; ^##^p < 0.01, ^#^p < 0.05 *vs.* MA + anaesthesia group.

### Paracetamol increases hippocampal anti-inflammatory cytokines following surgery.

Hippocampi were collected at the same time as plasma for the same cytokine analysis. Complete results are presented in Table 2. Briefly, Fisher’s LSD test revealed that MA animals which underwent surgery and were treated with Par-150 mg/kg had significantly higher levels of TNF-α compared to MA control or MA animals exposed to anaesthesia only (Table 2: p < 0.05). IL-13 levels were also significantly increased in MA animals which underwent surgery and were treated with Par-75 mg/kg or Par-150 mg/kg compared to MA control and compared to MA animals exposed to anaesthesia only (Table 2: p < 0.01 for both). Moreover, IL-10 levels were significantly increased in MA animals which underwent surgery procedure and were treated with Par-150 mg/kg compared to MA animals exposed to anaesthesia only (Table 2: p < 0.05).

**Table 2:** Analysis of cytokine levels in hippocampal tissue.

Hippocampus cytokines	MA control	MA + anaesthesia	MA + surgery model (Bup-0.05 mg/kg)	MA + surgery model (Bup-0.1 mg/kg)	MA + surgery model (Par-75 mg/kg)	MA + surgery model (Par-150 mg/kg)	F value
**Pro-inflammatory**							
TNF-α	100 ± 11 n = 5	101 ± 9.6 n = 8	104 ± 13 n = 5	92 ± 6 n = 6	108 ± 11 n = 8	141 ± 11*^#^ n = 8	F_(5, 34)_ = 2.643 p = 0.0402
IFN-γ	100 ± 6 n = 8	100 ± 6 n = 9	103 ± 6 n = 5	109 ± 20 n = 5	109 ± 5 n = 9	112 ± 6 n = 10	F_(5, 41)_ = 0.4818 P = 0.7877
IL-1β	100 ± 2 n = 8	102 ± 1 n = 9	102 ± 3 n = 5	104 ± 3 n = 6	103 ± 2 n = 10	105 ± 2 n = 11	F_(5, 44)_ = 0.5981 p = 0.7015
IL-6	100 ± 8 n = 8	108 ± 9 n = 10	108 ± 9 n = 5	93 ± 10 n = 5	96 ± 7 n = 7	98 ± 11 n = 10	F_(5, 40)_ = 0.3838 p = 0.8569
IL-5	100 ± 3 n = 8	97 ± 2 n = 7	Below Detection	100 ± 2 n = 6	102 ± 3 n = 9	104 ± 2 n = 9	F_(5, 36)_ = 0.6622 p = 0.6544
KC/GRO (CXCL1)	100 ± 4 n = 8	92 ± 3 n = 8	97 ± 8 n = 4	Below Detection	100 ± 8 n = 7	103 ± 2 n = 8	F_(5, 31)_ = 0.6642 p = 0.6533
**Anti-inflammatory**							
IL-10	100 ± 16 n = 9	84 ± 7 n = 10	91 ± 18 n = 5	97 ± 15 n = 6	118 ± 18 n = 9	127 ± 16 ^#^ n = 11	F_(5, 45)_ = 1.234 p = 0.3093
IL-13	100 ± 8 n = 7	112 ± 6 n = 10	94 ± 16 n = 5	126 ± 12 n = 6	164 ± 17**^##^ n = 7	162 ± 16**^##^ n = 10	F_(5, 39)_ = 4.828 p = 0.0016
IL-4	100 ± 12 n = 8	92 ± 7 n = 10	86 ± 6 n = 5	80 ± 4 n = 6	106 ± 12 n = 10	101 ± 7 n = 11	F_(5, 45)_ = 0.9694 p = 0.4467

Data are expressed as percentage of MA Control group ± SEM, n = 5–11 per group. One-way ANOVA followed by Fisher’s LSD analysis **p < 0.01, *p < 0.05 *vs.* MA control; ^##^p < 0.01, ^#^p < 0.05 *vs.* MA + Anaesthesia group.

### Hippocampal α-tubulin PTMs are altered following exposure to anaesthesia or surgery.

**Figure 5:**
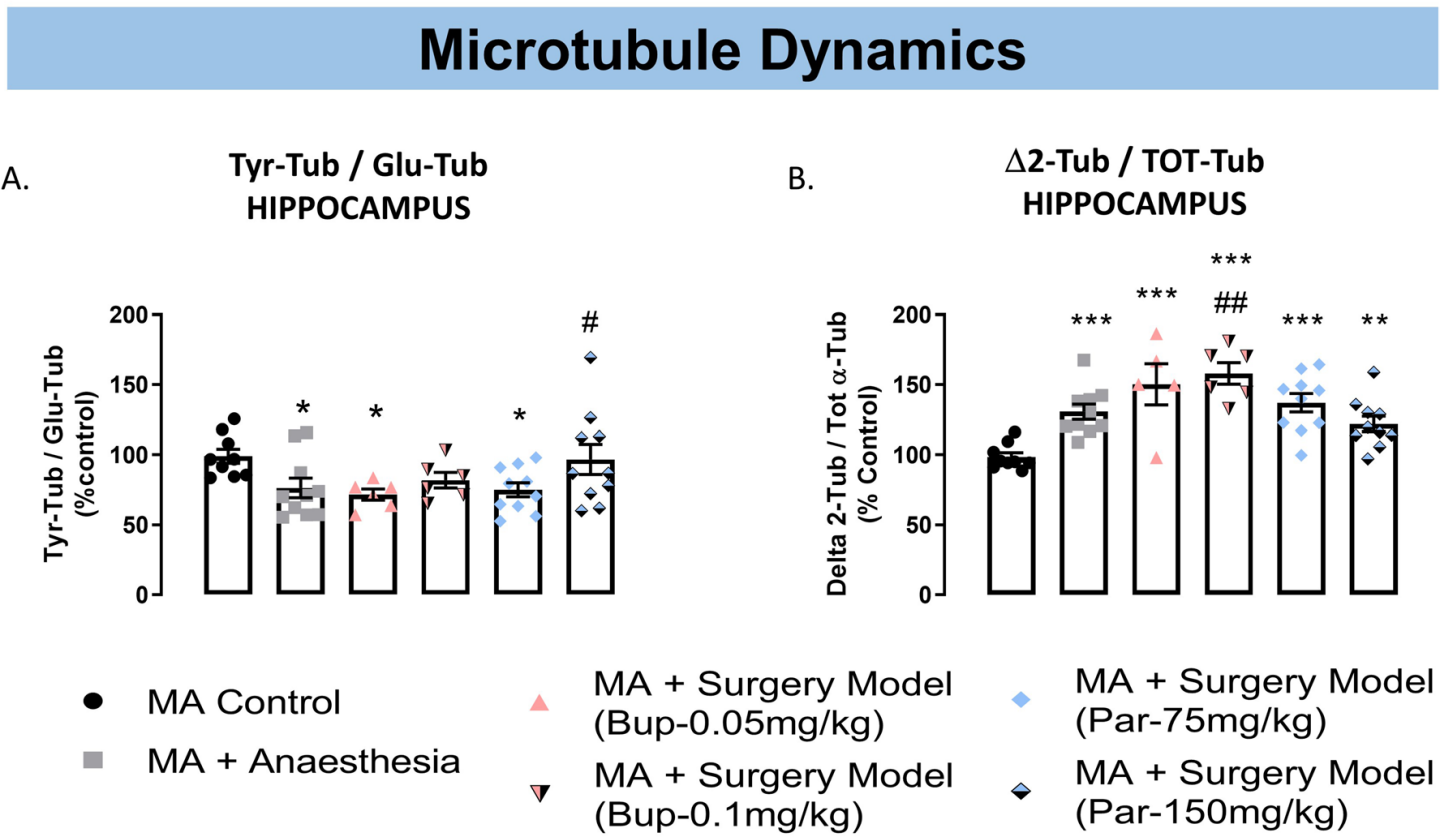
Hippocampal α-tubulin PTMs are altered following exposure to anaesthesia or surgery. (**A**) Tyr-Tub/Glu-tub ratio is decreased in MA animals exposed to anaesthesia only and in the surgery model groups treated with Bup-0.05 mg/kg or Par-75 mg/kg compared to MA control. (**B**) Δ2/TOT-Tub ratio is increased in MA animals exposed to anaesthesia only and in the surgery model groups treated with Bup-0,05 mg/kg or Bup-0.1 mg.kg (s.c.) or Par-75 mg/kg or Par150mg/kg (i.p.) compared to MA control. Surgery animals treated with Bup-0.1 mg/kg had significantly higher Δ2/Tot-Tub ratio compared to MA animals exposed to anaesthesia only. Histograms represent data expressed as a percentage of the MA Control group (mean ± SEM), n = 10–12 per group. One-way ANOVA followed by Fisher’s LSD analysis ***p < 0.0001, **p < 0.01, *p < 0.05 *vs.* MA control; ^#^p < 0.01, ^##^p < 0.01 *vs.* MA + Anaesthesia group.

α-tubulin PTMs were measured in the same hippocampus samples used for cytokine analysis. Expression of Δ2-Tub was normalized on expression of total α-tubulin (TOT-Tub), while Tyr-Tub and Glu-Tub were analysed as Tyr-Tub/Glu-Tub ratio. Analysis by one-way ANOVA of Tyr-Tub/Glu-Tub ratio yielded no main significant group effects (F_(5,45)_ = 2.15, p = 0.075). However, Fisher’s LSD pairwise comparisons revealed a significant decrease in Tyr-Tub/Glu-Tub ratio with anaesthesia exposure (p < 0.05) or surgery model procedure coupled with Bup-0.05 mg/kg (p < 0.05) or Par-75 mg/kg (p < 0.05) treatment compared to MA Control (Fig. 5A). The expression of Δ2-Tub/Tot-Tub ratio was also changed following anaesthesia or surgery model (one-way ANOVA; F_(5,44)_ = 7.6, p < 0.0001). Fisher pairwise comparisons revealed a significant increase in Δ2-Tub/TOT-Tub ratio with anaesthesia exposure (p < 0.01) or surgery model animals treated with Bup-0.05 mg/kg (p < 0.0001) or Bup-0.1 mg/kg (p < 0.0001) and surgery model animals treated with Par-75 mg/kg (p < 0.0001) or Par-150 mg/kg (p < 0.01), compared to MA Control (Fig. 5B). Finally, Δ2-Tub/Tot-Tub ratio was significantly increase in surgery model animals treated with Bup-0.1 mg/kg compared to MA animals exposed to anaesthesia only (Fig. 5B: p < 0.01).

## Discussion

### Paracetamol prevents cognitive decline and exerts long-lasting analgesic effects in experimental POCD

Our pilot study validated the DNMTS task protocol and confirmed previous findings, demonstrating that age-related working memory impairments in this task are evident in MA rats compared to YG controls^20^. The translational relevance of the DNMTS task is confirmed by the extensive use of delayed-response tasks in the clinic to identify age-related deficits in humans^21^. Here, we also showed that cognitive decline is evident in the DNMTS task at 48 h post-surgery in MA animals exposed to isoflurane alone or animals which underwent a surgery procedure and were treated with Bup-0.05 mg/kg (s.c.) or Bup-0.1 mg/kg (s.c.) as analgesia. Interestingly, animals which were treated with Par-75 mg/kg (i.p.) or Par-150 mg/kg (i.p.) were protected from POCD.

Previous studies have demonstrated that exposure of aged rats or mice to isoflurane alone was sufficient to induce spatial memory impairments^22,23^. Moreover, tibia fracture surgery in YG mice (12–14 weeks) using Bup (0.1 mg/kg, s.c.) as analgesia exhibited reduced freezing to context when compared with naive mice in a delay fear conditioning paradigm^2,3^. Aged mice (18 months) who underwent laparoscopy surgery with Bup (0.1 mg/kg, ip) as analgesia treatment displayed reduced cognitive flexibility when tested 24 h after surgery^24^. In one of the only other POCD studies carried out in rats, the authors report cognitive decline in aged rats (18-20mths) following tibia facture surgery with buprenorphine (0.3 mg/kg, ip) analgesia in the contextual fear-conditioning test and the Y-maze when tested at 24 h, 72 h and 7 days post-surgery^5^. We report temporary cognitive decline at 48 h post-surgery which appears to have recovered by the test at 72 h post-surgery. However, at the test on day 7 post-surgery, the animals exposed to anaesthesia alone have a tendency towards decreased performance in the DNMTS task and the animals treated with Bup-0.1 mg/kg are significantly impaired, whereas the animals treated with Par-150 mg/kg are comparable to the MA control group not exposed to anaesthesia or surgery. It is important to note, animals were maintained on a diet with restricted access to food. Previous studies have demonstrated weight reduction in rats by caloric restriction, before ischemic stroke model, in aged animals was associated with improvement in spatial memory in the Morris water maze test^25^. This reduction in weight was associated with decreased adipose tissue mass, circulating insulin, IGF1, and free fatty acids (FFA) levels which maybe linked to the recover of spatial memory compared to the non-calorie restricted group^25^. Here, all animals were maintained on the same diet regime, with anaesthesia exposed animals losing a similar amount of weight as those exposed to the surgical procedure (percentage weight loss: MA + Anaesthesia: 3.05% ; MA + Surgery (Bup-0.05 mg/kg): 3.6%; MA + Surgery (Bup-0.1 mg/kg: 4.3%; MA + Surgery (Par-75 mg/kg): 4.4%; MA + Surgery (Par-150 mg/kg): 4.9%).

It is noteworthy that a large number of the animals treated with Bup-0.05 mg/kg or Bup-0.1 mg/kg performed significantly less trials in the DNMTS task compared to the control group or animals exposed to isoflurane only. As a result, 7 animals were excluded from the Bup-0.05 mg/kg group and 6 animals were excluded from the Bup-0.1 mg/kg group. These side effects of buprenorphine treatment may be attributed to appetite suppression following Bup treatment^26^ resulting in reduced motivation to perform the DNTMS task. These data suggest buprenorphine may have a range of effects impacting motivation, reward and memory ultimately having a global negative effect on cognition. It cannot be excluded that animals treated with Bup may have experienced pain at the time of DNMTS testing as drugs were always administered 23 h before testing to avoid possible acute effects on cognition. Thus, our data from the cold plate test, which was carried out after the DNMTS task at approx. 24 h after drug administration, showed that surgery animals treated with Bup had increased sensitivity in the cold plate test compared to MA controls.

In contrast, the paracetamol treated surgery animals were not statistically different to the MA controls, suggesting that Par is a more effective long-lasting analgesic compared to Bup. Previous studies corroborate our observations since it was shown that single Bup (0.05 mg/kg, s.c.) administration was effective as post-operative analgesic in rats up to 4 h post administration^27^, while a single dose of Par (50–100 mg/kg, p.o.) has been shown to have anti-nociceptive effects up to 6 h post-administration^28^.Taken together, our findings are consistent with clinical studies in a young population (age from 26 to 41: mean = 36 years) showing that repeated administration of Bup at high dose (32 mg, oral admin, 10 days) had negative effects on memory resulting in delay-induced verbal memory impairment^29^. In addition, repeated administration of Bup at low dose (7 mg, oral admin, 18–28 weeks daily) was also associated with delayed recall of verbal information in opioid-addicted patients (mean age = 36 years)^30^. Remarkably, and again in line with our data, a single Par administration (2 mg, oral admin) was shown to improve performance in an information sampling task and increase hippocampal-based spatial memory in a double-blind clinical trial^31^.

### Paracetamol modulates inflammatory cytokines in the plasma and hippocampus

The current study analysed for the first time a larger panel of pro-inflammatory and anti-inflammatory cytokines protein levels in both plasma and hippocampus at day 8 after orthopaedic surgery. Our data show that anaesthesia alone did not affect systemic and central levels of any of the analysed cytokines, which is in line with previous reports^3^. IL-6 levels were increased only in the plasma and in the Bup-0.1 mg/kg surgery group, while IL-1β was unchanged in all groups in either plasma or hippocampus.

Previous studies reported increased plasma and hippocampal levels of IL-6 and IL-1β at 6 h and 24 h after receiving orthopaedic surgery and a single injection of Bup (0.1 mg/kg, s.c.) as analgesic in YG adult (3–4 months) mice^2,3^, but not at 2 h or 72 h after surgery^2^. Our study appears to confirm that plasma increase in IL-6 and IL-1β is also not a sustained event in an MA rat model of orthopaedic POCD, since it is not detectable at 8 days after surgery, with the exception of plasma IL-6 levels that were significantly increased in animals receiving a daily dose of Bup-0.1 mg/kg (s.c.). This latter observation is consistent with work showing increased plasma IL-6 levels following isoflurane anaesthesia and Bup administration before (0.025 mg/kg, s.c.) and after (0.05 mg/kg, s.c.) a scald-burn procedure^32^. Other authors observed increased plasma and synovial IL-6 levels after knee joint injury in mice injected with Bup (0.1 mg/kg, s.c.) followed by administration of the drug in drinking water for another 24 h^33^. The Bup-induced increase in plasma IL-6 levels might be linked to potential adverse effects of Bup as shown by our DNMTS task data.

An increase in plasma TNF-α has been previously shown to be rapid and transient, since it appears at 30 min after orthopaedic surgery following a single injection of Bup (0.1 mg/kg, s.c.) as analgesic, but not at 1 h, 2 h, 6 h and 12 h after surgery) in young adult (3–4 months of age) mice^3^. Additionally, protein levels of TNF-α have been reported to be increased in the prefrontal cortex of aged mice (20 months of age) at 6 days after surgery^4^. It is hypothesised that that circulating TNF-α plays an important role in POCD and that it reaches the brain, following surgery, via physiological penetration of the blood–brain barrier^3^ and disruption of the blood–brain barrier associated both with cognitive impairment and inflammatory response has been reported following tibia fracture^34^. Additionally, TNF-α is produced and released in the brain predominantly by microglia, astrocytes and neurons^35^. It has been speculated that increased brain levels of TNF-α might be involved in cognitive decline in brain disorders via potentiation of glutamate excitotoxicity (reviewed in^36^).

Intriguingly, our results show that 8 days after surgery animals that received Bup present cognitive deficits and no altered levels of hippocampal TNF-α, while animals treated with Par-150 mg/kg have no cognitive deficit but increased hippocampal TNF-α. Therefore, TNF-α appears to play a different role in the long-term cognitive deficits observed in our orthopaedic surgery models of POCD. It is of note that TNF-α has been shown to physiologically modulate Hebbian synaptic plasticity and synaptic scaling in the hippocampus where it can exert both excitotoxic or neuroprotective effects. For example, pre-treatment of hippocampal slices with TNF-α after hypoxia improved LTP in the DG^37^, while overexpression of TNF-α in transgenic mice results in potentiation of LTP in CA1 region^38^. In the central nervous system, Par can be converted into N-arachinodyl-phenolamine (AM404)^39^, which is an inhibitor of the anandamide membrane transporter (AMT) and therefore indirectly increases anandamide levels and stimulate CB1 receptors^40^. It has been shown that CB1 receptors activation reduces the TNF-α-mediated potentiation of striatal spontaneous glutamate-mediated excitatory postsynaptic currents^41^.

Since the behavioural data of this study shows that Par prevents cognitive decline in POCD, it is possible to speculate that the observed sustained central increase in TNF-α might keep the correct synaptic plasticity homeostasis, and that its potential glutamate-induced excitotoxicity might be reduced by the indirect activation of the CB1 receptors induced by AM404. Additional experiments are required to investigate this speculative hypothesis in the future. On the other hand, TNF-α promotes fracture repair in both rodent models and in clinical settings^42^. Thus, the increase in circulating levels of TNF-α in the Par treated group can be beneficial in promoting a rapid repair of the tibial damage caused by the orthopaedic surgery employed in our POCD model. Importantly, this hypothesis appears to be corroborated by the long-lasting analgesic effects we have observed in the Par treated animals compared to Bup.

Increased circulating levels of the pro-inflammatory IL-5 and the anti-inflammatory IL-13 have been observed in our study 8 days after surgery in MA rats receiving Bup in a dose-dependent pattern, but not in animals that received Par. Both cytokines are secreted peripherally and their increase is associated with lung allergic reaction^43,44^ and drug hypersensitivity^45^. Thus, our results may be linked to an adverse systemic reaction to the repeated Bup treatment. In contrast with the plasma data, we observed a significant increase of IL-13 in the hippocampus following Par administration (at both doses), but not following Bup. There is no evidence that IL-13 can pass the BBB, but some experimental studies showed its local production in the CNS by microglia and neurones and a potential neuroprotective role (reviewed in Mori^46^). Thus, IL-13 can be produced by neuronal cells in the hippocampus and the cortex in models of ischemic insult where it induced an alternative activation of microglia, exerting a protective effect against neuronal damage^47^. Hence, it is possible to speculate that the observed increase in hippocampal IL-13 levels may have neuroprotective effect.

IL-10 is systemically produced and plays a critical role in preventing inflammatory and autoimmune pathologies by limiting the release of pro-inflammatory cytokines^48^. As for IL-13, we showed that IL-10 is increased in the plasma of animals receiving Bup-0.1 mg/kg, but not Par; while the opposite is observed in the hippocampus. Our results are consistent with a previous study showing increased IL-10 serum levels following repeated Bup (0.075 mg/kg, s.c.) in a mouse model of arthritis^49^. Experimental models have shown that IL-10 is a “brain active” cytokine potentially produced in situ by microglial cells (for a review see^50^). IL-10 protects astrocyte from excessive inflammation by inhibiting the microglia production of pro-inflammatory cytokines^51,52^ and IL-10 receptor signalling has been associated with increased cellular survival and neurogenesis^53–55^. Therefore, the increase in hippocampal IL-10 observed following administration of the Par-150 mg/kg may have neuroprotective role, which is in line with the parallel increase of IL-13 and possibly that of TNF-α.

### Paracetamol modulates microtubule dynamics in the hippocampus

Previous studies have shown persistent alterations in hippocampal synaptic plasticity in experimental rodent models of POCD^5,6^, but microtubule dynamics has never been investigated. Here we have analysed hippocampal α-tubulin PTMs resulting from the cycle of detyrosination/tyrosination and associated with microtubule dynamics (i.e. Tyr-Tub, Glu-tub and Δ2-Tub) at 8 days after orthopaedic surgery. Specifically, the detyrosination/tyrosination cycle of α-tubulin consists of the enzymatic removal of the C-terminal tyrosine the re-addition of the tyrosine residue^56,57^ resulting in Glu-Tub (detyrosinated α-tubulin) and Tyr-Tub (tyrosinated α-tubulin, respectively), which are here analysed as a Tyr-Tub/Glu-Tub ratio^58,59^. High levels of Glu-Tub are found in stable microtubules, while dynamic microtubules express more Tyr-Tub^60–62^. Additionally, Glu-Tub can be converted into a stable, entity which cannot re-enter the cycle, named Δ2-Tub by removal of the last glutamate residue^63^. In the brain, Δ2-Tub is principally expressed in neuronal cells where it appears restricted to very stable microtubules^63^.

Our results showed for the first time decreased Tyr-Tub/Glu-Tub in MA rats exposed to isoflurane alone or in animals which underwent orthopaedic surgery procedure and were treated with Bup-0.05 mg/kg or Bup-0.1 mg/kg and Par-75 mg/kg. Previous studies have shown that cognitive deficits induce by a rat model of social isolation are paralleled by decreased Tyr-Tub/Glu-Tub in the hippocampus^64^ and rescued by drugs having pro-cognitive efficacy^58^. Recently, the clinical link between Tyr-Tub/Glu-Tub and cognitive decline has been proposed based on post-mortem studies carried out in the hippocampus of Alzheimer disease patients showing alterations in the detyrosination/tyrosination cycle of α-tubulin^65^. Furthermore, our results show that the observed increased expression of Glu-Tub (i.e. decreased Tyr-Tub/Glu-Tub ratio) is accompanied by significant increase in Δ2-Tub production in all experimental groups. Interestingly, such an increase in Δ2-Tub is more pronounced in animals that received Bup following orthopaedic surgery which is suggestive of neuronal accumulation of this irreversible α-tubulin PTM due to overexpression of Glu-Tub induced by Bup over time.

Lack of Tubulin Tyrosine Ligase (TTL; the enzyme that produces Tyr-Tub) in mice leads to brain accumulation of Glu-Tub and Δ2-Tub, impairment of the cortico–thalamic loop caused by abnormal neuronal projections, and alterations of neurite, dendrite and axon formation in primary neuronal cell culture^66^. It has been proposed that neuronal abundance of Glu-Tub and Δ2-Tub may lead to hyper-stabilization and altered interaction with MAPs eventually resulting in major impairments in axonal and dendritic formation (for a review see Janke^67^). Consistently, sustained changes in markers of synaptic plasticity have been reported in experimental models of POCD. Specifically, aged mice (16 months of age) that underwent laparotomy exhibited long-term cognitive decline paralleled by increases in neuroapoptotic markers (i.e. caspase-3 and iNOS) and decreased neuronal plasticity markers (i.e. BDNF, PSD-95 and synapsin-1) 7 days after surgery^6^. In the only other POCD studies carried out in rats and using a similar orthopaedic surgery, aged rats (18–20 months of age) showed long-term cognitive deficits accompanied by increased apoptosis and AMPAR GluA2 internalization 7 days after surgery^5^. It was also observed that 2 h inhaling exposure to the anaesthetic sevoflurane, compared to infusion of propofol, precipitated the reported surgery-induced synaptic changes^5^. Therefore, our results on the C-terminal detyrosination/tyrosination cycle of α-tubulin are in line with previous literature on synaptic alteration in POCD and further strength the possibility of a neuroprotective efficacy of Par administration compared to other analgesic such as Bup.

### Paracetamol has potential neuroprotective efficacy: a new avenue for the treatment of POCD

Our data showed for the first time that Par has potential neuroprotective efficacy following orthopedic surgery as a model of POCD in MA rats. Thus, Par administration: (i) prevented post-operative cognitive impairment in the operant DNMTS task, (ii) exerted long-lasting analgesic properties in the cold plate test; (iii) modulated circulating (i.e. plasma) and central (i.e. hippocampus) inflammatory cytokines; and (iv) increased hippocampal microtubule dynamics as indicated by alteration in α-tubulin PTMs expression. Taken all together, these findings support the use of Par as potential first-choice analgesic in POCD in clinical settings as an alternative to opioids such as Bup^10^. Furthermore, our data also open the path to exciting research projects focused on studying the potential neuroprotective efficacy of Par.

## Materials and methods

### Animals

Middle aged (14–16 months old, 473 ± 3 g on arrival) Male Sprague Dawley rats sourced from Envigo UK, were used in these experiments. The animals were pair-housed in a controlled environment (temperature: 20–22 °C, 12/12 h light/dark cycle (lights on at 8 a.m.)), with water ad libitum. Animals were maintained on a restricted diet during training, with a minimum of 85% of free-feeding weight. Once animals had learned the DNMTS task, they were fed 50 g/kg lab chow at the end of the experimental day which allows animals to gain weight but still have motivation to perform the task daily. Animals were acclimatized to the facility environment for 2 week prior to starting experiments. All experiments were performed under license from the Health Products Regulatory Authority (HPRA) of Ireland in accordance with EU regulations and with local ethical approval (the University of Dublin, Trinity College Dublin) as well as with the ARRIVE guidelines. Rats were chosen for this study as rats are the preferred species for studies involving complex cognitive measures as cognitive effects. Rats are superior at preforming tasks involving high-level strategy and show more stable performance in longer cognitive tests. Mice exhibit less strategy, needing substantially more training and practice to cognitive tasks and experience more stress and anxiety while doing so^68^.

### Drug administration

Par was administered at two doses [75 mg/kg and 150 mg/kg: dissolved in vehicle solution 1 (0.5% Methylcellulose in 0.9% saline)] and administered by the intraperitoneal (i.p.) route (see Minville et al.^69^ for chosen dose). Bup was administered at two doses [0.05 mg/kg and 0.1 mg/kg: dissolved in vehicle solution 2 (saline 0.9%)] by s.c. (see Zhang et al.^70^ for chosen doses). Control animals were treated with both vehicle 1 and vehicle 2. The Bup group were also treated with vehicle 1 while the Par group received additional control treatment of vehicle 2. All treatments were administered approx. 23 h before testing in the DNMTS task. Therefore, the acute effects of the compounds did not interfere with performance in the DNMTS task. Treatments were administered daily from the day of surgery and every day until animals were euthanized.

### Delayed non-match to sample (DNMTS) training protocol

DNMTS was performed as previously described^20^. Specifically, the rats were initially habituated to the operant conditioning chambers with the three levers extended. The animals were trained for 2 days to lever press for food reward on a continuous reinforcement schedule (i.e. pressing of any lever would result in the delivery of a sucrose pellet to the hopper). On the subsequent 2 days the levers were programmed to retract once pressed, delivering a pellet and then extending again. This was also on a continuous reinforcement schedule aimed to habituate the animals to the retraction and extension of the levers. On day 5, the same program was used with the exception that one specific lever could not be reinforced more than 3 consecutive times. This modification was aimed to force the animals to perform alternate lever pressing, thereby suppressing lever preferences to obtain reward.

The next phase of training involved randomised presentation of the front lever (left or right) and once pressed the extension of the back-lever was triggered. The reward was delivered only after the back lever was pressed. These lever combinations were repeated 60 times (30 left/center and 30 right/center) at 10 s intervals; this procedure was repeated for 2 days.

Training in the non-match-to-sample task was comprised of 90 trials in a maximum 90 min session daily. At the start of each session the house light is on with the levers in the retracted position. The animals were initially trained on the task contingencies with no enforced delay between the sample and the choice component (0-delay condition). At the start of each trial one response lever was randomly selected and inserted into the chamber (the “sample”). As soon as the lever press response was registered the lever was retracted and the rear lever on the opposite wall extended. Once the response on the back lever was registered the two front levers were extended into the chamber together (the “choice”). If a correct response was registered (i.e. a response on the non-matching to sample lever) the levers retract and a pellet delivered to the hopper, the house light remained on and an inter-trial interval of 10 s was initiated before the next trial began. If an incorrect response was registered (i.e. a response on the initial sample lever) no pellet was delivered, the house light extinguished and the 10 s interval initiated before the next trial started. Rats were required to meet a criterion of 85% for 3 consecutive days on this program before introduction of the delay. In the next stage of training a randomised 1 to 5 s delay was introduced between the response on the sample lever and the extension of the rear lever. This phase lasted for 3 days.

In the final stage of training, a random delay of 1–30 s was introduced requiring the rat to wait for the extension of the rear lever before moving to the choice phase. Training continued on this phase of the task until the animals’ performance reached a plateau (40 sessions).

*Testing* Following surgery, animals were allowed to recover for 48 h before resuming testing in the DNMTS task. Animals were treated with Vehicle or Analgesia 23 h before test in the DNMTS task to avoid potential acute drug effects interfering with task performance. Animals were tested each day from 48 h post-surgery/control procedure up to and including day 8 post-surgery. Each daily session was composed of 90 trials of different delay lengths. Completion of the 90 trials each day was used as a control for any potential drug-induced interference in task performance. As such any animal which did not complete the 90 trials each day was not included in the analysis of DNMTS data. MA Control and MA + Anesthesia groups each had one animal removed leaving n = 10 for each group. The MA + Surgery Model (Bup-0.05 mg/kg) had 7 animals excluded, leaving n = 5. The MA + Surgery Model (Bup-0.1 mg/kg) had 6 animals excluded, leaving n = 6. The MA + Surgery Model (Par-75 mg/kg) had 3 animals excluded, leaving n = 9. The MA + Surgery Model (Par-150 mg/kg) had 1 animal excluded, leaving n = 11. Bup treatment may have effects the animal’s ability to perform the task.

### Surgery model: tibia fracture with intra-medullary fixation

This procedure was performed as described^71,72^ and adapted for rats^5^. The procedure was performed by an experienced surgeon under supervision of the Designated Veterinarian (DV) at Trinity College Dublin. Induction and maintenance of anesthesia monitoring was carried out. Rats were placed in an induction box with 5% isoflurane. The left hind paw was shaved and sterilized with surgical scrub. Rats were placed in a facemask and on a heat pad and maintained under isoflurane anesthesia at 2–3%. Rats received one dose of their respective drug treatment (depending on treatment group) prior to surgery following anaesthesia with isoflourane. An incision was made in the surgical area and an appropriately sized pin (width 0.25-mm) inserted into the medullary canal. The wound was sutured and the rat was placed in a recovery cage on a heating pad before being returned to the home cage. Rats were treated with analgesia as indicated per group (Bup (0.05 mg/kg or 0.1 mg/kg) or Par (75 mg/kg or 150 mg/kg)) following surgery and once daily for the remaining duration of the study. All animals which underwent the surgery model procedure received analgesia as it would be unethical to perform this procedure without analgesia.

### Plasma and tissue collection

Animals were sacrificed following completion of the last test in the DNMTS task and brain tissue and blood was collected. Trunk blood was collected immediately in lithium heparin tubes and centrifuged at 200G for 15 min at room temperature. The platelet-rich plasma was then removed and placed into an Eppendorf and spun again at 2100G, 4 °C for 10 min, the plasma is then transferred to another Eppendorf, with 2% protease inhibitor cocktail (P8340, Sigma) and frozen at − 80 °C. Brains were immediately extracted and the hippocampi were removed and placed into Eppendorfs, snap frozen on dry ice and stored at − 80 °C until use.

### Infrared Western Blot (IFWB)

Hippocampus samples were processed for IFWB analysis as previously described^57^. Briefly, samples were adjusted to 0.02 μg for microtubules analysis and 0.2 μg total synaptic markers of total protein concentration per 1 μl with v/v Laemmli buffer 2× (Sigma). Electrophoreses were performed using 26-well 10% Bisacryliamide/Trisacrylamide gels (NuPAGE, Invitrogene) and 7.5 μl of each sample loaded. IFWB was performed with primary antibodies against Acet-Tub (clone 6-11B-1, Sigma) diluted 1:10,000, Total-Tub (clone DM1A, Sigma) diluted 1:15,000, Tyr-Tub (clone TUB-1A2, Sigma) diluted 1:20,000, Glu-Tub (polyclonal, Sigma) diluted 1:4000, Delta2 (polyclonal, Sigma) diluted 1:10,000, PSD-95 (clone 7E3-1B8, Sigma) diluted 1:10,000, Synaptophysin (clone SY38, Abcam) diluted 1:10,000. Membranes were incubated with an anti-mouse IRDye 680 or anti-rabbit IRDye 800 (LiCor) secondary antibody diluted 1:5000. The infrared signal was detected using the Odyssey scanner (LiCor).

### Multiplex cytokine analysis

Hippocampal samples were removed from − 80 °C and homogenised by sonication in lysis buffer (490 µl and 1:50 protease inhibitor, P8340, Sigma). Bradford assay was performed to prepare all samples to a common protein concentration. Prior to multiplex analysis plasma samples were defrosted and diluted (1:2) and standardized hippocampal samples by dilution (1:8). Cytokine levels for rat plasma/hippocampal samples were determined in duplicate using a V-Plex Multi-Spot assay system (pro-inflammatory panel 2 (Rat)) by Meso Scale Diagnostics. Plates were read using the MESO QuickPlex SQ 120 instrument and analyzed by Discovery Workbench 4.0 software.

### Statistical analysis

DNMTS trials were sorted by performance according to length of delay on individual trials and were grouped according to 5-s interval time bins (1–5, 6–10, 11–15, 16–20, 21–25, and 26–30). The data are presented graphically by total correct responses or percentage of correct responses at each 5-s delay time bin. All DNMTS data were statistically analyzed with SPSS, using t-test or RM ANOVAs and Fisher’s pairwise comparisons. Molecular data were analysed by one-way ANOVA followed by Fisher’s pairwise comparisons. GraphPad prism was used for graphical representations and figures were prepared in Office PowerPoint 16.
